# Investigation of Human *IFITM3* Polymorphisms rs34481144A and rs12252C and Risk for Influenza A(H1N1)pdm09 Severity in a Brazilian Cohort

**DOI:** 10.3389/fcimb.2020.00352

**Published:** 2020-07-10

**Authors:** Jéssica S. C. Martins, Maria L. A. Oliveira, Cristiana C. Garcia, Marilda M. Siqueira, Aline R. Matos

**Affiliations:** ^1^Laboratório de Vírus Respiratórios e do Sarampo, Instituto Oswaldo Cruz (Fiocruz), Rio de Janeiro, Brazil; ^2^Laboratório de Desenvolvimento Tecnológico em Virologia, Instituto Oswaldo Cruz (Fiocruz), Rio de Janeiro, Brazil

**Keywords:** influenza, biomarker, IFITM3, polymorphism, susceptibility

## Abstract

Influenza is a major public health problem that causes acute respiratory infection in humans. Identification of host factors influencing in disease outcome is critical for recognition of individuals with increased risk. Investigations on the role of rs34481144A and rs12252C *IFITM3* polymorphisms in influenza A(H1N1)pdm09 severity is not yet conclusively determined. This study aimed to evaluate such polymorphisms frequencies and *IFITM3* levels in an infected Brazilian cohort of 314 influenza A(H1N1)pdm09 cases and its putative association with clinical, epidemiological and virological data. Individuals were clinically classified into mild, severe and fatal cases. *IFITM3* polymorphisms were detected by specific Taqman probes in real time PCR reactions. *IFITM3* levels were determined by quantitative real time PCR. Thus, the different clinical groups presented similar distribution of rs34481144 and rs12252 genotypes and allelic frequencies. There was no significant association between the polymorphisms with severity of disease by using distinct genetic models. Additionally, geographic distribution of mutants showed that rs34481144A allele was more predominant in Brazilian Southern region. In contrast, rs12252C allele presented similar frequencies in all regions. Individuals with the distinct rs34481144 and rs12252 genotypes showed similar levels of *IFITM3* and viral load in their respiratory specimens. Furthermore, *IFITM3* levels were comparable in the distinct clinical groups and were not correlated with influenza viral load in analyzed samples. Thereby, rs34481144A and rs12252C polymorphisms were not associated with severity or mortality of influenza A(H1N1)pdm09 infection nor with *IFITM3* transcript levels and influenza viral load in upper respiratory tract samples in a Brazilian cohort.

## Introduction

Influenza viruses cause acute respiratory infections in humans, which are correlated to annual epidemics and occasional pandemics, representing a relevant public health problem (Taubenberger and Morens, 2008). Annually, an estimated one billion cases of influenza, of which 3–5 million are severe cases, result in 300,000–650,000 deaths, worldwide (Danielle Iuliano et al., 2018; World Health Organization, 2019). Children under 5 years old, the elderly, individuals with chronic diseases and immunocompromised are more prone to develop severe disease, composing the standard risk groups for this infection (Cox and Subbarao, 1999). Influenza A(H1N1)pdm09 and H3N2 subtypes, as well as B viruses, are the current major sources of human influenza infections. Currently, vaccination and antiviral treatment are the main control measures of this infection. However, due to the viral evolutionary patterns, vaccines must be updated regularly (Epperson et al., 2019). In addition, identification of influenza strains that are resistant to the current antiviral drugs has been reported, yet still in low levels (Matos et al., 2018; Takashita et al., 2020).

Because influenza A virus infect distinct animal hosts, genetic shift can eventually occur, arising genetic and antigenically distinct reassortant viruses. Depending on their ability of inter-individual transmission within the human susceptible population, these emerging viruses pose a potential pandemic risk, which occurred in 2009, with the introduction of influenza A(H1N1)pdm09 virus into the human population. During this period, healthy young adults were severely affected and counted for a significant portion of the fatalities (Taubenberger and Morens, 2008).

The clinical evolution of influenza infection can be influenced by multiple factors, including individual susceptibility, pathogen virulence and host pre-existing antibody titers. Influenza virulence can be altered by acquisition of mutations during virus evolutionary process that can modify its interactions with the infected cell, increasing viral affinity for cell receptors (Chutinimitkul et al., 2010; Liu et al., 2010), and by influencing host immune response (Fislová and Kostolanský, 2005). Likewise, viruses can evade immune response conferred by antibodies produced during previous infections and by vaccination (van der Sandt et al., 2012). Similarly, host genetic variants may also affect modulation of immune response after viral infection (Falcon et al., 2015; Chan et al., 2017). Putative associations between genetic polymorphisms in host crucial genes that act against virus infection that are essential for virus-host interplay have been recently explored regarding their association with susceptibility/severity of influenza infections (Zhou et al., 2012; Tarnow et al., 2014; Cheng et al., 2015; Chan et al., 2017; Garcia et al., 2018; Matos et al., 2019).

Interferon-induced transmembrane protein three (IFITM3) is a proinflammatory cytokine belonging to the group of interferon (IFN) stimulated genes (ISGs), which are induced after viral infection. The process of induction of ISGs starts following the fusion of viral envelope with the cell endosomal membrane, when the viral genetic material is released into the cytoplasm and allows its recognition by cellular pattern recognition receptors (PRRs) (Heil et al., 2004; Kawai and Akira, 2010; Kim et al., 2016) that stimulate secretion of type I IFNs, which, in turn, bind to their receptor (IFNAR) and activate the JAK/STAT pathway, driving ISGs expression. IFITM3 presents an influenza restriction function by preventing formation of fusion pores necessary for the release of viral genetic material into the cytoplasm, thus abrogating virus replication (Feeley et al., 2011; Huang et al., 2011; Suddala et al., 2019). Complementarily, it has been described that *ifitm3* knockout mice show higher levels of influenza replication and develop fulminant viral pneumonia (Everitt et al., 2013).

Because of its central role in influenza restriction (Brass et al., 2009; Feeley et al., 2011; Everitt et al., 2013; Desai et al., 2014), single nucleotide polymorphisms (SNPs) in *IFITM3* gene have been associated with an increased severity of influenza A(H1N1)pdm09 infection (Randolph et al., 2017; Allen et al., 2018; Zani and Yount, 2018; Kim et al., 2019). One of the investigated SNPs, the rs34481144A, leads to a substitution in *IFITM3* promoter region (Randolph et al., 2017) and a further modification of a methylation site. These events increase the affinity for the CTCF transcriptional factor, which could interfere with *IFITM3* transcription. The presence of this mutation was previously associated with lower *IFITM3* expression levels and decreased binding affinity for the regulatory factor IRF3. A previous relationship of this SNP with higher risk of severe influenza infection has been described (Allen et al., 2018). In addition, the rs12252C is described as responsible for generating a truncated protein, lacking the initial 21 amino acids of the N-terminal region (Δ21 IFITM3) (David et al., 2018). The deleted region comprises the regulatory YEML internalization motif recognized by the AP-2 complex, that conducts IFITM3 localization into late endosomes, multivesicular bodies and lysosomes (Chesarino et al., 2014; Jia et al., 2014). Moreover, the PPNY motif, also included in the deleted region, recruits NEDD4 to promote IFITM3 ubiquitination and turnover via lysosomes (Chesarino et al., 2015). Therefore, rs12252C modifies IFITM3 intracellular localization and levels which, consequently, interfere with restriction against influenza virus (Everitt et al., 2013; Compton et al., 2016). Despite that, some studies have shown that patients that present rs12252C in homozygosity displayed the majority of *IFITM3* transcripts as the complete isoform (Randolph et al., 2017; Makvandi-Nejad et al., 2018). However, the association of these polymorphisms and the risk of severe influenza is not totally clear (Zhang et al., 2013; Kim and Jeong, 2017; Pan et al., 2017; Randolph et al., 2017; David et al., 2018).

In this study, we report the distribution of *IFITM3* rs34481144A and rs12252C polymorphisms in a Brazilian cohort of influenza A(H1N1)pdm09 positive cases, whose samples were collected during the 2012–2018 period, further classified according to their clinical presentation as mild, severe and fatal infections. Furthermore, we explored putative relationships between these SNPs with clinical, epidemiological and virological variables, in addition to *IFITM3* expression levels.

## Materials and Methods

### Population

Our laboratory is a National Reference Laboratory part of the Influenza Surveillance System (ISS) for the Brazilian Ministry of Health and is one of the National Influenza Centers (NICs) of the World Health Organization (WHO) (WHO, 2020). Thus, we systematically receive samples from nine out of 27 Brazilian states (Southern, Northeastern and Southern regions). During the 2012–2018 period, approximately 25,000 influenza A(H1N1)pdm09 cases were investigated by the Brazilian ISS. Almost 10% of these samples were received at our NIC (Fiocruz, Rio de Janeiro). In this study, we included 314 respiratory clinical samples that were collected from each patient as three individual swabs from the two nostrils and the oropharynx. In this type of samples, we mostly detect epithelial cells (Daley et al., 2006). Inclusion criteria consisted of the availability of clinical and epidemiological records, which were collected by ISS teams from each Brazilian State. Further, cases were classified into influenza-like illness (ILI), severe acute respiratory infection (SARI) and fatal cases. ILI was defined as presence of fever (even if reported) and cough or sore throat, plus one of the following symptoms: headache, myalgia or arthralgia. SARI cases were defined as cases requiring hospitalization and presenting dyspnea or one of the following signs of severity: peripheral capillary oxygen saturation <95%, respiratory distress or acute respiratory insufficiency (Secretaria de Vigilância em Saúde, 2012). This study was approved by Fiocruz-IOC Ethics Committee, approval number 2.453.470.

### Influenza Detection

Total RNA was extracted from clinical respiratory samples by using the QIAmp Viral RNA Mini kit (Qiagen, Germany), according to the manufacturer's instructions. For influenza A(H1N1)pdm09 detection, extracted RNA was tested in a one-step real time RT-PCR assay using specific primers and probes (CDC, USA), as recommended by the WHO. Influenza viral load was indirectly determined by real-time RT-PCR at the moment of diagnosis as the CT (cycle threshold) number during its detection (Behillil et al., 2018; Mares-Guia et al., 2020).

### Genotyping

Genomic DNA was extracted from clinical respiratory samples by using the PureLink™ Genomic DNA Mini Kit (Invitrogen, USA), as previously described (Garcia et al., 2018; Matos et al., 2019). After that, the rs34481144A and rs12252C polymorphisms were identified using pre-defined TaqMan^TM^ SNP Genotyping Assays probes (assays IDs: C__26288451_10 and C_175677529_10) (Thermo Fisher Scientific, USA) in a real time PCR assay performed in the Step One Plus equipment (Applied Biosystems, USA) with 5–20 ng DNA, per sample, according to manufacturer's instructions. Cycling parameters were as follows: 95°C for 10 min, followed by 50 cycles of 95°C for 15 s and 60°C for 90 s. In this methodology, each TaqMan probe presents a distinct fluorescent signal and is specific for amplification of a different nucleotide at each position. In the end of the run, the software groups the samples according to their specific unique (homozygotes) or mixed (heterozygotes) fluorescence signal, as previously described (Randolph et al., 2017; Garcia et al., 2018).

### Quantification of *IFITM3* Expression

After total RNA extraction, we proceeded to reverse transcription with oligo (dT) primers and Superscript III Reverse Transcriptase (Thermo Fisher Scientific, USA). Quantitative real time PCR was carried out in a real time PCR Step One Plus equipment (Applied Biosystems, USA) with the following specific primer sequences for *IFITM3*: F−5′ GGTCTTCGCTGGACACCAT 3′ and R−5′ TGTCCCTAGACTTCACGGAGTA 3′. All reactions were conducted using the SYBR Green detection reagent (Applied Biosystems, USA) and were carried out in duplicates. Cycling conditions consisted of the initial steps of 50°C for 2 min and 94°C for 5 min, followed by 40 cycles of 94°C for 30 s, 50°C for 30 s and 72°C for 45 s. A final melting curve analysis was performed to determine amplification specificity (60–95°C with a heating rate of 0.2°C/s and continuous fluorescence measurement). Expression data were normalized to glyceraldehyde 3-phosphate dehydrogenase (GAPDH) expression and analyzed by using the 2^−ΔΔ*CT*^ method.

### Statistical Analysis

The Hardy-Weinberg equilibrium (HWE) for the allele frequency and genotype distribution was assessed by using the web program (http://www.oege.org/software/hwe-mr-calc.shtml) (Rodriguez et al., 2009), and deviation from HWE was assessed by Chi-square test. After descriptive analyses, putative associations between outcomes (genotypes) and clinical/epidemiological traits were explored (Chi-square or Fisher's exact test for discrete variables and ANOVA for means) using SPSS software for Windows, version 19.0 (IBM Inc., USA). Additional statistical analysis was performed with GraphPad Prism (GraphPad Software Inc., USA). *IFITM3* expression level and influenza viral load values, in the clinical or genotyped groups, were compared by Student's *T*-test or One-Way ANOVA with Bonferroni post-test for multiple comparisons. Finally, correlation between *IFITM3* expression and influenza viral load were assessed by linear regression to calculate R^2^ and *p*-values. Results were considered significant when *p* < 0.05.

## Results

### Clinical, Demographical and Epidemiological Characteristics of the Enrolled Brazilian Patients

In this study, we investigated 314 patients positive for influenza A(H1N1)pdm09 infection during the 2012–2018 period. Individuals were classified according to their infection severity as ILI (*n* = 92), SARI (*n* = 140) or fatal (*n* = 82) cases, as mentioned in the methodology section. Of note, influenza A(H1N1)pdm09 virus was the most prevalent subtype among the Brazilian cases from this period (data from Brazilian ISS, available at http://portalms.saude.gov.br/saude-de-a-z/gripe/situacao-epidemiologica-dados). Therefore, 51.6% of the patients were male and the median age of the individuals was 39 years old (Table 1). Moreover, the median age of the patients in the ILI, SARI and fatal groups were significantly different (29, 43, and 46 years old, respectively; *p* < 0.01), as already expected, since the higher the age, the greater the risk of disease complications (Cox and Subbarao, 1999). Among SARI patients, 88.1% presented dyspnea, 71.3% respiratory distress and 46.1% oxygen saturation < 95%. Also, 53.1% of the patients informed any comorbidity (pneumopathy, cardiopathy, immunodepression, hypertension, hepatopathy, neurologic disorders, nephropathy, diabetes, cancer, obesity and smoking), and they were less predominant in the ILI group, as compared to SARI and fatal groups (24.3, 59.6, and 63.6%, respectively; *p* < 0.01), considering that comorbidities are also risk factors for complications. Regarding demographical origin of the samples, most of them were collected at the Brazilian Southern region, followed by the Southeastern and the Northeastern regions (52.9, 30.9, and 16.2%, respectively; *p* < 0.01; Table 1).

**Table 1:** Clinical, demographical and epidemiological characteristics of the influenza A(H1N1)pdm09 Brazilian patients whose samples were included in this study.

**Characteristics**	**Total**	**ILI**	**SARI**	**Fatal**	***p*-value**
Number of samples	314	92	140	82	NA
Age (years)—median ± SD	39 ± 22.6	29 ± 18.5	43 ± 24.8	46 ± 20.7	<0.01
Male—n (%)	162 (51.6)	52 (56.5)	63 (45.0)	47 (57.3)	0.11
**Signs and symptoms**					
Cough—n/N (%)	246/255 (96.5)	66/69 (95.6)	138/140 (98.6)	42/46 (91.3)	0.06
Sore throat—n/N (%)	90/199 (45.2)	38/64 (59.4)	43/105 (41.0)	9/30 (30.0)	0.01
Coryza—n/N (%)	21/161 (13.0)	14/58 (24.1)	5/76 (6.6)	2/27 (7.4)	<0.01
Dyspnea—n/N (%)	165/184 (89.7)	NA	119/135 (88.1)	46/49 (93.9)	0.25
Respiratory distress—n/N (%)	111/160 (69.4)	NA	82/115 (71.3)	29/45 (64.4)	0.39
Oxygen saturation <95%—n/N (%)	77/160 (48.1)	NA	53/115 (46.1)	24/45 (53.3)	0.40
Comorbidities—n/N (%)	95/179 (53.1)	9/37 (24.3)	65/109 (59.6)	21/33 (63.6)	<0.01
**Geographic region**					
South—n (%)	166 (52.9)	67 (72.8)	49 (35.0)	50 (61.0)	<0.01
Southeast—n (%)	97 (30.9)	9 (9.8)	67 (47.8)	21 (25.6)	
Northeast—n (%)	51 (16.2)	16 (17.4)	24 (17.1)	11 (13.4)	

*Statistical significance was considered when p < 0.05. All comparisons were performed between the clinical groups. SD, standard deviation; ILI, influenza-like illness; SARI, severe acute respiratory infection; NA, not applicable; n, number of cases with the referred characteristic; N, number of cases for which the information is available*.

### Relationship of *IFITM3* Polymorphisms rs34481144A and rs12252C and Severe Influenza A(H1N1)pdm09

**Figure 1:**
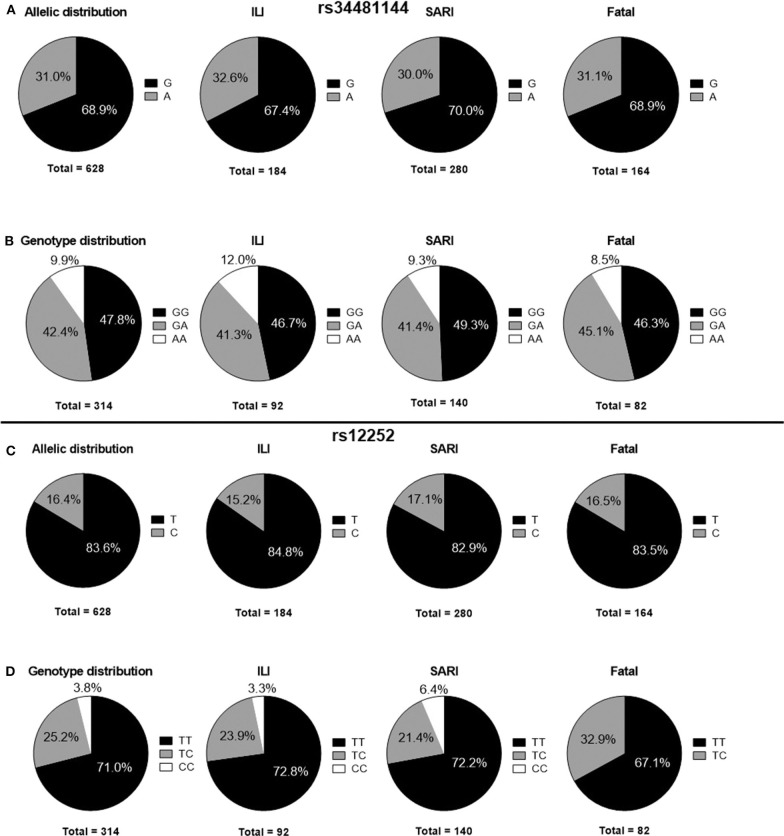
Distribution of *IFITM3* polymorphisms in ILI, SARI and fatal cases. **(A)** rs34481144 allelic distributions. **(B)** rs34481144 genotype distributions. **(C)** rs12252 allelic distributions. **(D)** rs12252 genotype distributions. ILI, influenza-like illness; SARI, severe acute respiratory infection.

In our Brazilian cohort, the frequencies of *IFITM3* rs34481144A and rs12252C genotypes were in accordance with HWE (*p* > 0.05). The overall *IFITM3* rs34481144A allelic frequency was 31.0% (Figure 1A). The distribution of rs3448144 genotypes was as follows: 47.8% of the individuals had the GG wild type (WT), whereas 42.4% had the GA heterozygous genotype and 9.9% had the AA mutant homozygous genotype (Figure 1B). There was no difference in the distribution of rs34481144 genotypes and alleles between the ILI, SARI and fatal groups, when using additive, dominant, recessive and allelic genetic models (*p* = 0.92, *p* = 0.88, *p* = 0.71, and *p* = 0.83, respectively; Table 2). Distinct stratification of the clinical groups (ILI vs. SARI + fatal or ILI + SARI vs. fatal) also failed to identify association of rs34481144A with severity or mortality.

**Table 2:** Distinct models for the risk assessment of *IFITM3* rs34481144 and rs12252 genotypes.

**rs34481144**					**rs12252**				
**Additive**	**ILI**	**SARI**	**Fatal**	***p*-value**	**Additive**	**ILI**	**SARI**	**Fatal**	***p*-value**
**Genetic model**									
AA—n (%)	11 (12.0)	13 (9.3)	7 (8.5)	0.92	CC—n (%)	3 (3.3)	9 (6.4)	ND	0.88^*^
AG—n (%)	38 (41.3)	58 (41.4)	37 (45.1)		CT—n (%)	22 (23.9)	30 (21.5)	27 (32.9)	
GG—n (%)	43 (46.7)	69 (49.3)	38 (46.4)		TT—n (%)	67 (72.8)	101 (72.1)	55 (67.1)	
**Dominant**					**Dominant**				
AG + AA—n (%)	49 (53.3)	71 (50.7)	44 (53.7)	0.88	CT + CC—n (%)	25 (27.2)	39 (27.9)	27 (32.9)	0.65
GG—n (%)	43 (46.7)	69 (49.3)	38 (46.3)		TT—n (%)	67 (72.8)	101 (72.1)	55 (67.1)	
**Recessive**					**Recessive**				
AA—n (%)	11 (12.0)	13 (9.3)	7 (8.5)	0.71	CC—n (%)	3 (3.3)	9 (6.4)	ND	0.73^*^
GG + AG—n (%)	81 (88.0)	127 (90.7)	75 (91.5)		TT + CT—n (%)	89 (96.7)	131 (93.6)	82 (100)	
**Allelic**					**Allelic**				
A—n (%)	60 (32.6)	84 (30.0)	51 (31.1)	0.83	C—n (%)	28 (15.2)	48 (17.1)	27 (16.5)	0.86
G—n (%)	124 (67.4)	196 (70.0)	113 (68.9)		T—n (%)	156 (84.8)	232 (82.9)	137 (83.5)	

Statistical significance was considered when p < 0.05 and was assessed by chi-square test. All comparisons were performed between the clinical groups (ILI, SARI, and Fatal), with exception to where symbol

* * appears (ILI vs. SARI + fatal). ILI, influenza-like illness; SARI, severe acute respiratory infection; ND, not detected*.

Concerning the rs12252C polymorphism, we detected a total allelic frequency of 16.4% (Figure 1C). Rs12252 genotype distribution was 71.0% for WT (TT), 25.2% for heterozygous (CT) and 3.8% for mutant homozygous (CC) (Figure 1D). In a similar fashion to rs34481144, we did not observe differences in rs12252 genotypes and allelic distributions between the distinct clinical groups of influenza cases by assuming the distinct genetic models (Table 2). Noteworthy, the rare CC genotype was not identified in any of the fatal cases.

In our analysis, the rs34481144 and rs12252 alleles were not associated with age, gender, clinical symptoms and comorbidities, suggesting that their frequencies are not influenced by these factors.

Intriguingly, the presence of rs34481144-AA and rs12252-CC homozygous mutant genotypes concomitantly was not detected in any of the influenza cases in our cohort (*p* < 0.001). The rs3448114-AA recessive genotype was always transmitted with the rs12252-TT dominant genotype, and the rs12252-CC recessive genotype was ever inherited with the rs34481144-GG dominant genotype in our influenza cases. By contrast, both dominant genotypes (GG and TT) could be inherited with any of the other genotypes. These results suggest that their presence at homozygosity does occur simultaneously or consists of a rare event.

### Geographical Distribution of rs12252C and rs34481144A Polymorphisms

In Brazil, distinct geographical regions present dissimilar patterns of genetic ancestry due to specific migratory currents from distinct continents (Alves-Silva et al., 2000; Mychaleckyj et al., 2017). In the light of this knowledge, we investigated SNPs allelic and genotypes distribution in the influenza cases according to their geographical Brazilian region. Therefore, we observed that rs34481144A allelic prevalence was higher in the Brazilian Southern region, followed by the Southeastern and Northeastern regions (34.6; 29.4, and 22.5%, respectively; *p* = 0.05). In contrast, rs12252C allele presented similar patterns of predominance in the Southern, Southeastern and Northeastern regions (14.1, 19.1, and 37.7%, respectively; *p* = 0.29).

### Analysis of Association of Polymorphisms With *IFITM3* Expression and Influenza Viral Load

**Figure 2:**
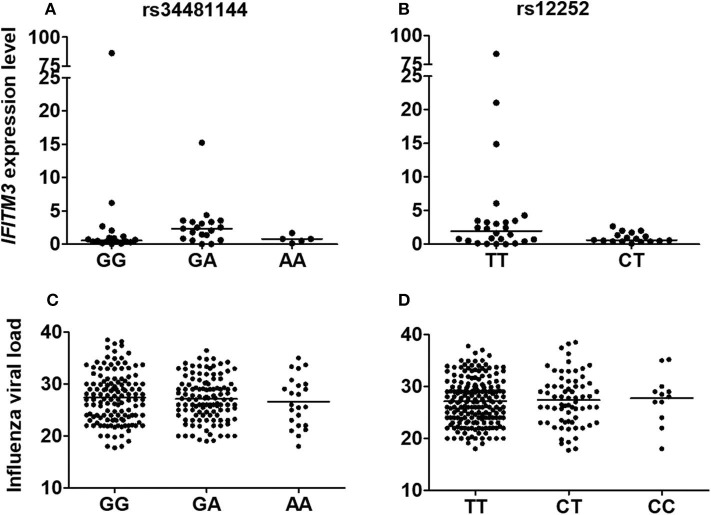
Relationships between rs34481144 and rs12252 genotypes with *IFITM3* expression levels [**(A,B)**, respectively] and with influenza A(H1N1)pdm09 viral load [**(C,D)**, respectively] in human respiratory clinical samples. *IFITM3* expression level was assessed by quantitative real time PCR (ΔΔCT method) by using GAPDH as a housekeeping gene. Influenza viral load was determined by real-time RT-PCR CT (cycle threshold) number during its detection. Bars represent median.

Because it was previously shown that rs34481144A and rs12252C could interfere with *IFITM3* expression, we analyzed its transcript levels in our genotyped influenza positive clinical specimens. As a result, *IFITM3* presented comparable levels among distinctly genotyped rs34481144 and rs12252 subjects (*p* = 0.69 and 0.20, respectively; Figures 2A,B). Of note, as rs12252-CC genotype was detected in a low frequency in our cohort (3.8%, Figure 1D), material was not enough to perform robust expression analysis.

Additionally, we further explored a possible correlation between rs34481144 and rs12252 genotypes and influenza viral load, but we did not detect any significant difference in our positive cases (Figures 2C,D). Furthermore, as IFITM3 can restrict the replication of influenza viruses (Brass et al., 2009; Everitt et al., 2013), we evaluated the correlation between IFITM3 levels and influenza viral load, regardless of the genotype. However, the analysis showed a poor correlation coefficient (*R*^2^ = 0.02; *p* = 0.34; Supplementary Figure 1A and Supplementary Table 1). Moreover, IFITM3 expression in the respiratory specimens showed no differential levels in the different clinical groups ILI, SARI and fatal (Supplementary Figure 1B).

## Discussion

The antiviral restriction factor IFITM3 acts as part of innate immune response initial steps to limit influenza infection. It halts the formation of viral membrane fusion pores with host endosomes, by clustering in the IAV containing endosomes, impairing entry into the cytosol and subsequent viral replication (Kummer et al., 2019; Suddala et al., 2019). In addition, IFITM3 has also been shown to be induced, in DCs, after influenza infection, in mice, and is involved in migration of these cells to lymph nodes and consequent activation of influenza-specific lymphocytes (Infusini et al., 2015). Additionally, resident lung CD8+ T memory cells present persistent higher levels of IFITM3, which protect these cells to secondary influenza infections (Wakim et al., 2013). Because of these pivotal roles during immune response, some authors have suggested that genetic variation in the *IFITM3* gene could affect its function. Some precedent studies have investigated rs34481144A and rs12252C distribution in the context of influenza infection and its clinical progression (Jia et al., 2012; Everitt et al., 2013; Allen et al., 2018).

Herein, we examined the frequency of such SNPs in Brazilian human clinical respiratory samples collected from influenza A(H1N1)pdm09 positive cases during the post-pandemic period, from 2012 to 2018. Although the frequency of rs34481144A in our influenza cohort (31,1%) was substantially higher than those previously described for healthy subjects from the Americas region (23.3%; Auton et al., 2015), the presence of this polymorphism was not associated with severe clinical outcomes nor with *IFITM3* expression levels and influenza viral load in respiratory samples. These findings suggest that SNP rs34481144A could even favor a higher susceptibility to viral infection, however, does not account for the development of severe viral infection. The results observed in our cohort are opposed to those reported by Allen et al., who found that rs34481144A was associated with severe influenza infection in three cohorts, as well as showed a reduction of *IFITM3* levels in PBMCs from A allele carriers (Allen et al., 2018). It is noteworthy to point out that the epithelial cells of the respiratory tract are the initial target of influenza infection, and therefore constitute a good model for assessing the level of expression of *IFITM3* during influenza infection. The different results found in our study and in the study by Allen et al. are probably due to the different cellular types used in the studies. Conversely, a different study described that rs34481144A had a protective effect against severe viral infection under the dominant model, as well as similar *IFITM3* promoter activity when comparing rs34481144 WT and mutant HeLa cells (David et al., 2018). This last study demonstrated that rs34481144A occurrence in Portuguese influenza positive individuals (31.1%) was highly similar to our Brazilian positive cohort (30.5%), additionally to a similar frequency of allele A between severely affected Brazilian patients (30.4%) and the Portuguese (22.7%), a likely result due to our deep historical relationship, as a former Portuguese colony. It also corroborates our results on the similarity of *IFITM3* expression, despite the genotype. Further functional studies in representative cohorts of different geographical areas, involving several cellular types and *in vitro* models are necessary to better elucidate the effective rs34481144A role in influenza infection.

Similarly, we found no association between the presence of rs12252C and clinical outcomes of influenza infection, *IFITM3* expression levels and influenza viral load in our specimens. In our study, the rs12252 CC genotype was not detected in patients in the fatal group, probably due to the low frequency of this genotype in our cohort. Earlier studies had detected a positive correlation between rs12252C and disease severity, mainly in Chinese cohorts (Zhang et al., 2015; Chan et al., 2017; Pan et al., 2017) whereas research conducted with others cohorts, such as Europeans, Americans, Afro-Americans and Korean failed to identify such association (Mills et al., 2014; Gaio et al., 2016; Kim and Jeong, 2017; Randolph et al., 2017). The overall allelic frequency of rs12252C in our Brazilian influenza A(H1N1)pdm09 cohort (16.4%) was lower than those reported for Chinese infected cohorts (69.4 and 65.7%; Zhang et al., 2013; Pan et al., 2017), but showed comparable frequency with a Portuguese cohort (9.8%; David et al., 2018), also reinforcing genetic inheritance similarities between Brazil and Portugal. The same trend was observed when rs12252C allelic frequency was compared in severely ill patients, as the Brazilian cohort (16.9%) has a similar prevalence as the Portuguese (9.1%), whereas is inferior to the Chinese ones (76.5 and 81.2%). Under a complementary perspective, rs12252C total allelic rate in an American healthy cohort (17.7%) is lower than an Eastern Asian one (53.0%; Auton et al., 2015). Therefore, a speculative explanation for this set of observations could be that allelic frequency of rs12252C is considerably higher among the Chinese population, when compared to others.

A very provocative finding of this report was that the recessive genotypes of both SNPs were not detected together and were always inherited with the dominant genotype of the other locus. This outcome was also previously described by others (Randolph et al., 2017; Allen et al., 2018; David et al., 2018). In addition, we showed that the geographical distribution of the rs34481144 and rs12252 alleles genotypes had opposed trends, as Brazilian infected individuals from the South presented the highest rs34481144A allele frequency (34.6%) but the lowest rs12252C one (14.1%). On a similar fashion, the lowest frequency of rs34481144A allele in the Northeast was matched with the highest rs12252C allele frequency. Moreover, the frequency of rs34481144A in healthy population from the Americas (23.0%) was higher than those found in Africa (4.0%) and Eastern Asia (1.0%). In contrast, rs12252C frequency in the Americas (17.7%) was lower than in Africa (26.%) and East Asia (53.0%) (Auton et al., 2015). Altogether, these observations regarding opposite inheritance also related to geographical trends suggest that these alleles are in a strong linkage disequilibrium (LD), as also pointed out by others (Randolph et al., 2017; Allen et al., 2018; David et al., 2018). The LD pattern of such *loci* could be affected by natural selection, genetic drift, recombination and mutation (Slatkin, 2008). However, once we did not observe a relationship between these SNPs and *IFITM3* expression levels in our analysis, further investigations need to be performed to better elucidate the functional correlation of these two *loci* inheritance patterns.

Considering the lack of connection between the genotypes and *IFITM3* expression, we further noted that IFITM3 transcript levels, in our respiratory specimens, were not correlated with influenza viral load nor with clinical status of the influenza cases. This observation would likewise suggest that *IFITM3* levels, in the moment of diagnosis, in this type of samples, are not related to the disease outcome and could not be used as a molecular biomarker for the disease progression.

In conclusion, our results showed no association of rs34481144A and rs12252C with severity or mortality of influenza infection nor with *IFITM3* transcript levels and influenza viral load in upper respiratory tract samples in the Brazilian infected cohort studied. Subsequent functional studies would add valuable information on the role of these SNPs during influenza infection.

## Data Availability Statement

The raw data supporting the conclusions of this article will be made available by the authors, without undue reservation, to any qualified researcher.

## Ethics Statement

The studies involving human participants were reviewed and approved by Fiocruz-IOC Ethics Committee. Necessity of written informed consent was avoided in this study because the participants were evaluated under the Brazilian ISS, as approved by the local Ethics Comittee.

## Author Contributions

JM and AM designed the experiments. JM, CG, and AM conducted the experiments. JM, MO, and AM performed the statistical analysis. JM, MO, CG, MS, and AM contributed to the writing and editing of the manuscript. AM and MS contributed with funding acquisition. All authors contributed to the article and approved the submitted version.

### Conflict of Interest

The authors declare that the research was conducted in the absence of any commercial or financial relationships that could be construed as a potential conflict of interest.

## Supplementary Material

Supplementary Figure 1 *IFITM3* expression level in human respiratory clinical samples. **(A)** Correlation analysis of *IFITM3* expression level and influenza viral load was performed by linear regression. R2 and *p*-value were calculated. Expression was assessed by quantitative real time PCR (ΔΔCT method). GAPDH was used as the housekeeping gene. **(B)** *IFITM3* expression level in clinical samples classified according to influenza severity as ILI, SARI and fatal cases. Bars represent median.

Supplementary Table 1 Data used for the correlation analysis of *IFITM3* expression level and influenza viral load. Influenza viral load was assessed as a measure of RT-PCR cycle threshold (CT) for detection of influenza A H1Pdm09 hemagglutinin (HA) gene and *IFITM3* relative expression level, for each sample.
