# Supplementary figures and images for: Investigation of Human IFITM3 Polymorphisms rs34481144A and rs12252C and Risk for Influenza A(H1N1)pdm09 Severity in a Brazilian Cohort
Source: Front Cell Infect Microbiol. 2020 Jul 10;10:352. doi: 10.3389/fcimb.2020.00352 (PMC7366732; doi:10.3389/fcimb.2020.00352)

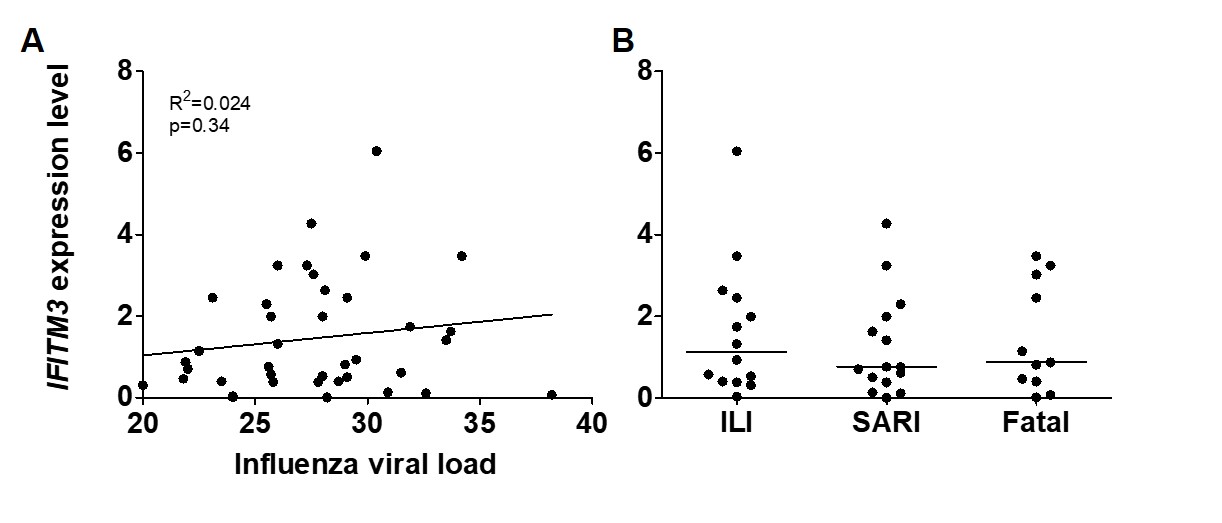

Supplement: Supplementary Figure 1 — IFITM3 expression level in human respiratory clinical samples. (A) Correlation analysis of IFITM3 expression level and influenza viral load was performed by linear regression. R2 and p-value were calculated. Expression was assessed by quantitative real time PCR (ΔΔCT method). GAPDH was used as the housekeeping gene. (B) IFITM3 expression level in clinical samples classified according to influenza severity as ILI, SARI and fatal cases. Bars represent median. [file Image_1.JPEG]
